# Quantitative Analysis and Predictive Modeling of Nonspecific Adsorption of Recombinant Adeno-Associated Virus onto Solid Surfaces

**DOI:** 10.1021/acs.langmuir.5c05368

**Published:** 2026-02-27

**Authors:** Yuki Ueda, Risa Shibuya, Koichi Shibata, Airi Murai, Yasuo Tsunaka, Mitsuko Fukuhara, Susumu Uchiyama

**Affiliations:** † Nissan Chemical Corporation, 5-1, Nihonbashi 2-Chome, Chuo-ku, Tokyo 103-6119, Japan; ‡ Department of Biotechnology, Graduate School of Engineering, 13013 The University of Osaka, 2-1 Yamadaoka, Suita, Osaka 565-0871, Japan; § U-Medico Inc., 2-1 Yamadaoka, Suita, Osaka 565-0871, Japan

## Abstract

Recombinant adeno-associated virus (rAAV) has been increasingly employed for *in vivo* gene therapy. To ensure the particle concentration meets specifications, strategies are necessary to minimize nonspecific adsorption of rAAVs onto solid surfaces during manufacturing and drug-product dispensing into vials. In this study, we first elucidated the physicochemical factors contributing to nonspecific adsorption of rAAVs by evaluating their adsorption on model surfaces with systematically controlled hydrophilicity and surface charge. Subsequently, we constructed a predictive model through multiple regression analysis of rAAV adsorption under various formulation conditions and the physicochemical parameters of both rAAV serotypes and investigated surfaces. The results revealed that both electrostatic and hydrophobic interactions were responsible for rAAV adsorption. Consequently, we designed a hydrophilic and near-neutral surface coating, which greatly suppressed the adsorption of multiple rAAV serotypes, even under the formulation conditions of marketed products, demonstrating the high versatility and predictive accuracy of the model. These findings validated the effectiveness of the surface modification strategy developed to ensure the stable manufacturing and quality control of rAAV products, offering a foundation for further material development toward clinical applications.

## Graphical abstract

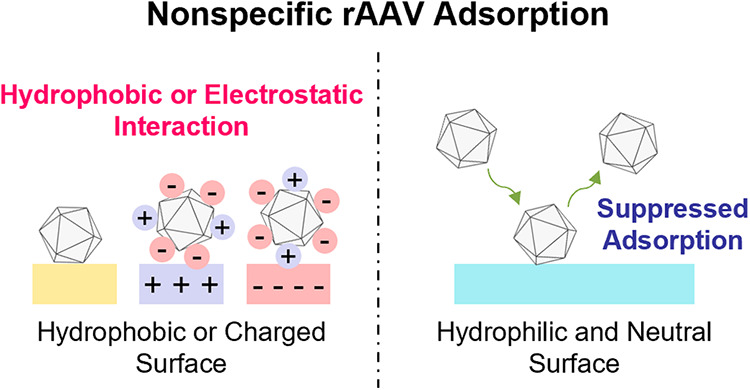

## Introduction

Recombinant adeno-associated virus (rAAV) exhibits minimal pathogenicity and very low frequency of integration into host genomes. rAAV has multiple serotypes, each exhibiting distinct tissue tropism, which enables efficient gene delivery to specific target organs and cells. These advantages have established rAAV as the leading platform for *in vivo* delivery of gene therapies.^1,2^ However, the high cost of rAAV-based products remains a major barrier to their widespread adoption. In addition to the high cost of manufacturing rAAV, significant challenges remain in developing cell lines for production, scaling up production, and improving purification processes.^3^

One of the existing issues encountered during the manufacturing and storage of rAAVs is the loss of rAAVs owing to nonspecific adsorption, which should not be underestimated because rAAV is typically handled at low concentrations. For example, the concentration of Zolgensma is approximately 30 nM (2.0 × 10^13^ vg/mL), whereas the typical concentration of a therapeutic antibody (10 mg/mL) is approximately 70 μM. Thus, adsorption of rAAV, which inevitably occurs in tubes, containers, and syringe needles, significantly impacts the vector concentration.^4^ In particular, nonspecific adsorption of rAAV onto solid surfaces, which occurs during storage and administration, can lead to considerable loss of valuable vectors and variability in administered doses. In an analysis of drug substances and drug products of rAAVs, inaccurate quantification is attributed to nonspecific adsorption of rAAVs to pipet tips and tubes in analytical instruments.^5^ Therefore, suppressing the nonspecific adsorption of rAAVs is critically important for stable manufacturing, quality control of rAAV products, and efficacy and safety of patients.

Nonionic surfactants such as poloxamer 188 (P188), polysorbate 20 (PS20), and polysorbate 80 (PS80) are widely used to minimize the nonspecific adsorption of proteins.^6^ However, these surfactants have several limitations. For example, when rAAV formulations are diluted before administration, the concentration of surfactants is also reduced, which generally weakens their ability to prevent adsorption. In addition, it has been reported that the effectiveness of surfactants can vary depending on the characteristics of the solid surface.^7–9^ A recent study investigating rAAV adsorption to various materials reported that the addition of P188 as a surfactant provides only limited suppression of rAAV adsorption onto glass surfaces.^10^ Furthermore, there are concerns regarding the stability and safety of surfactants. Polysorbates can degrade through hydrolysis and oxidation, and the resulting degradation products have been reported to affect the stability of formulations.^11,12^ Although P188 is less prone to degradation owing to its lack of unsaturated double bonds and ester linkages, it has been reported to undergo oxidative degradation, particularly in the presence of histidine buffer, which may accelerate its degradation.^13–16^ In addition, the degradation products of surfactants may cause injection site pain and hypersensitivity reactions,^14,17^ highlighting the need for careful evaluation during formulation design.

Against this background, there is a growing demand for technologies that suppress the nonspecific adsorption of rAAV without using surfactants or using minimal levels of surfactants. We recently reported that a coating composed of a polyionic hydrophilic complex (PHC) is effective in reducing the nonspecific adsorption of rAAVs onto solid surfaces.^5^ However, further studies are needed to gain a comprehensive understanding of the detailed mechanism of adsorption, which will improve the development of coating materials that minimize the adsorption of rAAVs.

Hydrophobic and electrostatic interactions are widely recognized as major driving forces of protein adsorption onto solid surfaces,^18–20^ and we hypothesize that these interactions also promote rAAV adsorption. To test this hypothesis, in this study, we prepared model surfaces with systematically controlled hydrophilicity/hydrophobicity and surface charge properties to elucidate the physicochemical factors contributing to the nonspecific adsorption of rAAVs. Owing to the predominant negative charges of rAAVs, a positively charged surface would promote electrostatic interactions, while hydrophobic interactions may vary depending on the rAAV serotype. In addition to surface properties, rAAV serotype and formulation composition were examined for their impact on adsorption. Furthermore, to construct a predictive model of rAAV adsorption, multiple linear regression analysis was performed to clarify the relationships between the measured adsorption ratio and physicochemical parameters of both solid surfaces and rAAVs. Specifically, the hydrophilicity/hydrophobicity and surface charge of each solid surface were determined by measuring the contact angle and zeta potential, respectively. Moreover, the hydrophilicity/hydrophobicity of each rAAV serotype was determined by calculating the solvent-accessible surface area and measuring the zeta potential. On the basis of the constructed model, we designed a new coating material to suppress the nonspecific adsorption of rAAVs and evaluated its performance under various formulation conditions.

## Materials and Methods

### Chemicals

KCl, KOH, and ethanol were obtained from Junsei Chemical Co. Ltd. (Tokyo, Japan). NaH_2_PO_4_·2H_2_O, Na_2_HPO_4_·12H_2_O, NaCl, HCl, and sucrose were obtained from FUJIFILM Wako Pure Chemical Corporation (Osaka, Japan). MgCl_2_·6H_2_O, mannitol, and tris­(hydroxymethyl)­aminomethane were obtained from Nacalai Tesque, Inc. (Kyoto, Japan). Poloxamer-188 (Kolliphor P 188 Bio) was kindly provided by BASF (Ludwigshafen, Germany).

### Preparation of rAAV Samples

rAAV samples were prepared using the method previously reported.^21,22^ Briefly, for rAAV2, rAAV8, and rAAV9, suspension-adapted HEK293F cells (Viral Production Cells 2.0, Thermo Fisher Scientific, Waltham, MA) were maintained in BalanCD HEK293 medium (FUJIFILM Irvine Scientific, Santa Ana, CA) supplemented with 6 mM l-glutamine (FUJIFILM, Tokyo, Japan). Cells were seeded at 1.0 × 10^6^ cells/mL, incubated (37 °C, 8% CO_2_, 130 rpm), and grown to a density of 2.0 × 10^6^ cells/mL for transfection. Cells of each serotype were cotransfected with a transgene plasmid, adenoviral helper plasmid, and Rep&Cap plasmid encoding capsid genes (rAAV2, rAAV8, and rAAV9). Plasmids were mixed at a 1:1:1 mass ratio (1 μg total DNA of rAAV2 and rAAV9 per 10^6^ cells and 0.75 μg of total DNA of rAAV8 per 10^6^ cells) and complexed with FectoVIR-AAV (Polyplus, Illkirch, France). Cultures of rAAV2 and rAAV9 were returned to the incubator and shaken for 72 h post-transfection. Cultures of rAAV8 were returned to the incubator and shaken for 96 h. For the rAAV5 cultures, HAT cells^22^ were maintained in HE400AZ medium (Gmep Incorporated, Fukuoka, Japan). Cells were seeded at 0.4 × 10^6^ cells/mL, incubated (37 °C, 8% CO_2_, 130 rpm), and grown to a density of 0.7 × 10^6^ cells/mL for transfection. Cells were cotransfected with a transgene plasmid, adenoviral helper plasmid, and Rep&Cap plasmid that encodes the capsid genes (rAAV5). Plasmids were mixed at a 1:1:1 mass ratio (total DNA, 1 μg per 10^6^ cells) and complexed with PEIpro (Polyplus, Illkirch, France). Cultures were returned to the incubator and shaken for 72 h post-transfection.

After transfection, both the medium and cell lysates were harvested. The lysates were clarified by centrifugation at 4000*g* for 20 min, followed by filtration through a 0.22 μm poly­(ether sulfone) filter (Sartorius, Goettingen, Germany). The resulting clarified lysates were applied to POROS GoPure AAVX prepacked columns (Thermo Fisher Scientific) equilibrated with Tris buffer containing 20 mM Tris, 0.001% P188, and either 0.15 M NaCl for rAAV5, rAAV8, and rAAV9, or 0.5 M NaCl for rAAV2. After column loading, impurities were removed by washing with 20 mM Tris buffer (pH 7.6) supplemented with 1 M NaCl and 0.001% P188. Bound rAAV particles were subsequently eluted under acidic conditions using 0.1 M citrate (pH 2.0–2.5), 0.4 M l-arginine (FUJIFILM, Tokyo, Japan), and 0.001% P188. Eluted fractions were immediately neutralized prior to further purification by density gradient ultracentrifugation (DGUC). For DGUC, the neutralized eluates were adjusted to a final CsCl concentration of 3 or 3.5 M (FUJIFILM, Tokyo, Japan) and centrifuged at 18,000–25,000 rpm for 24 h at 16 °C using a Beckman SW41Ti rotor (Beckman Coulter, Brea, CA) in an Optima XE-90 centrifuge. Fractions corresponding to full capsids were identified and collected using an online monitoring system, and the collected fractions were subsequently dialyzed against a buffer containing 200 mM NaCl and 0.001% P188 using Slide-A-Lyzer G2 or Slide-A-Lyzer G3 dialysis cassettes (Thermo Fisher Scientific).

### Preparation of Coating Materials and Coating Process

Hydrophilic surfaces were prepared using coating materials. Coating materials were prepared using previously reported methods.^23,24^ The coating polymers contained phosphate and amine units, enabling control of surface charge properties while maintaining hydrophilicity. Coating Neg (negatively charged) and Pos (positively charged) were initially prepared by adjusting the proportion of phosphate and amine units to evaluate rAAV adsorption to surfaces with different charges. On the basis of the results, Coating Neu (approximately neutral) was developed, and rAAV adsorption was assessed.

Polypropylene (PP) sheets (Johoku Co., Ltd., Tokyo, Japan) and PP microtubes (1.5 mL, 509-GRD-Q, Thermo Fisher Scientific) were coated and then used in the following surface property evaluations and adsorption experiments. Specifically, PP sheets were immersed in the coating solution for 5 min. The excess solution was removed, and then the sheets were dried at 90 °C for 24 h. The coated sheets were then washed with 70% aqueous ethanol and dried at 50 °C for 3 h to complete the coating process. For the microtubes, the coating solution was sprayed onto the inner surface using a spray coater. Then, the excess solution was removed, and the microtubes were dried and washed using the procedure applied to the coated PP sheets. Coated PP sheets and microtubes were denoted as Coating Neg/PP, Coating Pos/PP, and Coating Neu/PP.

### Characterization of Solid Surfaces

The hydrophilicity and surface charge properties of untreated and coated PP sheets were evaluated. Contact angle measurements were performed using a contact angle meter (DM-701, Kyowa Interface Science Co. Ltd., Saitama, Japan) in various solutions using the captive bubble method. An air bubble (2.0 μL) was introduced from below onto the solid surface immersed in the solution, and the static contact angle was determined through image analysis.

The zeta potential was measured using a zeta potential analyzer (SurPASS 3, Anton Paar GmbH, Austria). Untreated and coated PP sheets cut to 20 × 10 mm were mounted in the adjustable gap cell, and measurements were performed in various solutions. The pH of the 1 mM KCl solution (pH 7.4) was adjusted using a 10 mM KOH solution. The contact angle and zeta potential were each measured at least three times, and the average values were calculated.

### Characterization of rAAVs

To evaluate the surface charge properties of rAAV particles, the zeta potential was measured using a Zetasizer Ultra (Malvern Panalytical Ltd., Malvern, U.K.), disposable folded capillary cell (DTS1070), and solution containing 10 mM sodium phosphate buffer (pH 7.4), 50 mM NaCl, and 0.001% P188. The folded capillary cell was filled with 750 μL of the buffer and then loaded with 50 μL of the rAAV sample (2 × 10^12^ vg/mL), and measurements were performed. The zeta potential was calculated from the electrophoretic mobility using the Smoluchowski approximation, and the average value from three measurements was used for subsequent analysis. The net charge was estimated from the measured electrophoretic mobility and diffusion coefficient, as previously reported.^25^

To assess the structural characteristics of rAAV particles, the solvent-accessible surface area (SASA) of the capsid outer surface (hereafter referred to as outer SASA) was calculated by classifying residues as hydrophobic or hydrophilic components. SASA calculations were performed using Michel Sanner’s Molecular Surface (MSMS).^26^ To reflect the structural influence of neighboring viral proteins (VPs), the SASA of a partial capsid comprising multiple VPs was calculated, and the value corresponding to a single VP surrounded by neighboring VPs was extracted. To focus on atoms exposed on the outer surface of the capsid, only atoms located more than 105 Å from the capsid center were included. This threshold was determined through visual inspection using the molecular visualization software Chimera X. The following PDB entries were used for each serotype: AAV2 (PDB code: 1lp3),^27^ AAV5 (PDB code: 7kp3),^28^ AAV8 (PDB code: 2qa0),^29^ and AAV9 (PDB code: 3ux1).^30^ Residues were classified as hydrophobic residues (PHE, ILE, LEU, TYR, TRP, VAL, MET, PRO, CYS, and ALA) or hydrophilic residues (acidic: ASP and GLU, basic: LYS and ARG). The total side-chain SASA was then calculated for each category. Because a strong correlation (*r* = 0.93) was observed for SASA values of acidic and basic residues, they were combined and treated as a single explanatory variable in the regression analysis.

### Evaluation of Nonspecific Adsorption of rAAV Particles

To evaluate the nonspecific adsorption of rAAV particles, the adsorption of rAAVs onto the solid surfaces of untreated and coated PP microtubes was quantified as the adsorption ratio. The buffer solution used in the adsorption experiments was 10 mM sodium phosphate buffer (pH 7.4) containing 200 mM and 350 mM NaCl. The effect of surfactants was also investigated by using buffers with and without 0.001% P188 under the condition of 200 mM NaCl.

rAAV samples were dialyzed against the adsorption buffer containing 0.001% P188 and adjusted to a concentration of 5.5 × 10^12^ vg/mL. They were then diluted 100-fold with the same buffer, prepared with or without 0.001% P188 depending on the condition, and 200 μL aliquots were dispensed into untreated or coated PP microtubes. Although the dialysis buffer contained 0.001% P188, the final concentration became extremely low after 100-fold dilution with the surfactant-free buffer, and therefore this condition was regarded as surfactant-free. The tubes were then incubated at room temperature for 1 h. The same dilution procedure was performed just before the end of 1 h of incubation, and this sample was used as a preadsorption control. rAAV titers before and after adsorption were quantified by digital PCR, as described in the following section. All measurements were performed in triplicate. The adsorption ratio was calculated using the following equation:

adsorption ratio(%)=(titer before
adsorption−titer after
adsorption)/titer before adsorption×100

If the adsorption ratio was negative, it was considered a measurement error and treated as 0%.

To investigate the applicability of the constructed adsorption model under formulation conditions, as realistic cases, adsorption experiments were conducted using the formulation conditions of rAAV drug products. The formulation conditions of rAAV drug products^31–33^ were used for rAAV2, rAAV5, and rAAV9, while the optimal formulation condition reported in a recent study^34^ was used for rAAV8. Under these conditions, a buffer containing 0.001% P188 was used during dialysis. However, because the samples were diluted 100-fold, they were regarded as surfactant-free, the same condition as the sodium phosphate buffer. The detailed buffer compositions are shown in Table 2 (see Results and Discussion Section).

### Digital PCR Analysis

rAAV genomic titers were quantified by digital PCR using the QuantStudio Absolute Q Digital PCR System (Thermo Fisher Scientific), according to a previously reported method.^35^ Samples were treated with DNase I (Takara, Japan) at 37 °C for 30 min to digest unpackaged DNA, followed by the addition of 0.25 mM EDTA (Nippon Gene, Japan) and incubation at room temperature for 5 min. The reaction was then heated at 95 °C for 15 min to inactivate the DNase I enzyme and denature the viral capsid. Processed samples were diluted with Tris-EDTA buffer containing 0.001% P188 to adjust the concentration for analysis. Each digital PCR reaction was prepared to a final volume of 10 μL, consisting of 1 μL of sample, 2 μL of 5× Absolute Q Master Mix (Thermo Fisher Scientific), 1.8 μL of ITR primers (forward and reverse), and 0.25 μL of probe mix (Hokkaido System Science, Japan). The following primers and probe were used: ITR forward primer 5′-GGAACCCCTAGTGATGGAGTT-3′; ITR reverse primer 5′-CGGCCTCAGTGAGCGA-3′; and ITR probe 5′-[FAM]-CACTCCCTCTCTGCGCGCTCG-[BHQ1]-3′. Subsequently, 9 μL of the reaction mixture was added to each well of a QuantStudio Absolute Q MAP16 Plate Kit (Thermo Fisher Scientific), followed by the addition of 15 μL of QuantStudio Absolute Q Isolation Buffer (Thermo Fisher Scientific). The wells were sealed, and thermal cycling was performed on the system with the following conditions: 96 °C for 10 min, followed by 40 cycles of 94 °C for 5 s and 54 °C for 30 s. Data and global thresholds were analyzed using QuantStudio Absolute Q digital PCR software (Thermo Fisher Scientific).

### Multiple Regression Analysis

Multiple regression analysis was conducted to quantitatively evaluate the contributions of physicochemical parameters to the adsorption ratio. Python 3.12.2 and the statsmodels library (version 0.14.4) were used for the analysis, and the model was constructed using the ordinary least-squares (OLS) method. To obtain comparable standardized regression coefficients, all variables were standardized using *z*-scores.

## Results and Discussion

### Characterization of Solid Surfaces

The nonspecific adsorption of proteins is known to be driven by hydrophobic and electrostatic interactions.^18–20^ In this study, we investigated whether these interactions also promoted the adsorption of rAAV particles by preparing solid surfaces with systematically controlled hydrophilic/hydrophobic characteristics and surface charges. The hydrophilicity/hydrophobicity and electrostatic properties of PP, Coating Neg (high proportion of phosphate units)/PP and Coating Pos (high proportion of amine units)/PP were evaluated by measuring the contact angle and zeta potential, respectively (Figure 1). The contact angle of PP was 90°, and its zeta potential was −85.9 mV, indicating a hydrophobic and negatively charged surface. Previous studies have reported that neutral polymers, including PP, exhibit negative zeta potentials.^36,37^ This behavior is considered to result from the preferential adsorption of OH^–^ ions in the Stern layer of the electrical double layer.^36–38^ In contrast, Coating Neg/PP and Pos/PP showed a reduced contact angle of 30°, indicating increased hydrophilicity. The zeta potential of Coating Neg/PP surface was −61.7 mV, while the zeta potential of Coating Pos/PP surface was +37.2 mV. These coatings thus exhibited different surface charges but the same hydrophilicity for systematic evaluation of rAAV adsorption.

**Figure 1:**
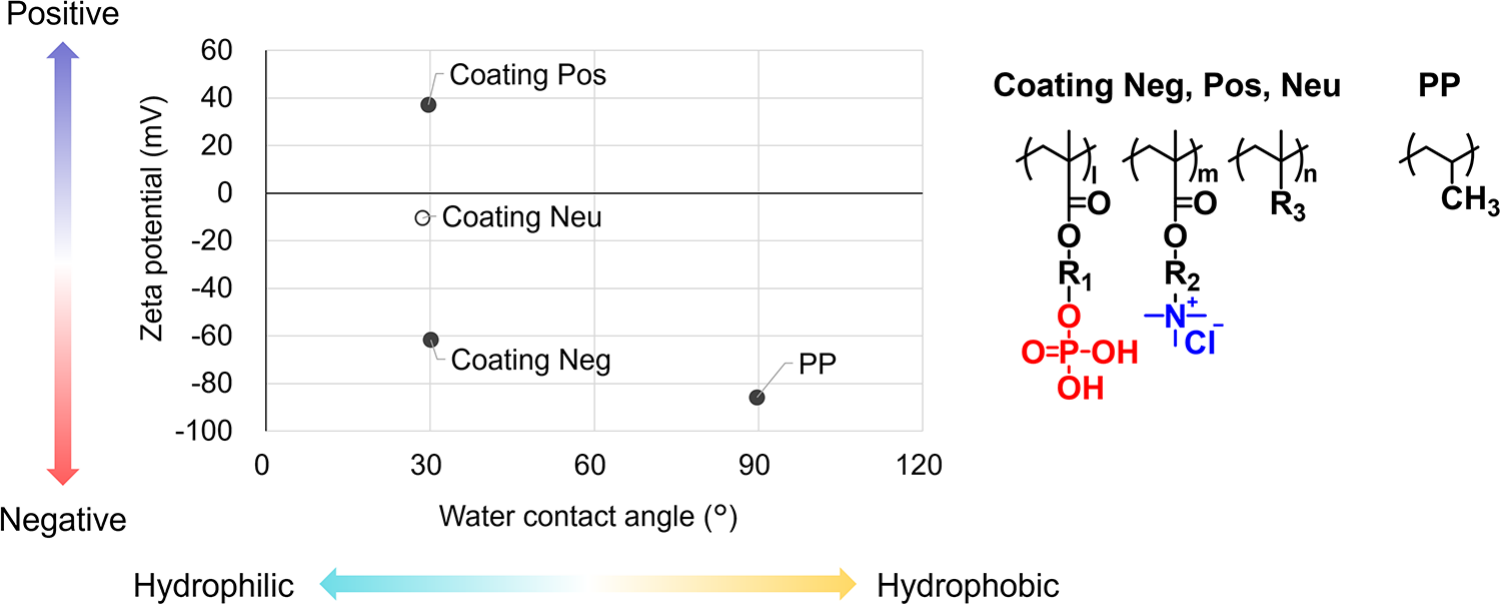
Water contact angle and zeta potential of the surfaces of PP, Coating Neg/PP, Coating Pos/PP, and Coating Neu/PP. The water contact angle was measured using the captive bubble method in pure water, and the zeta potential was measured in 1 mM KCl at pH 7.4. Chemical structures of PP and coatings are also shown. Coating Neg, Pos, and Neu have a common polymer backbone but varying proportions of phosphate and amine units. R_1_, R_2_, and R_3_ represent nonionic organic groups composed of carbon, hydrogen, and oxygen.

### Evaluation of Surface Charge and SASA of rAAV Particles

The surface charge of rAAV particles is considered to have a significant effect on their electrostatic interactions with solid surfaces. Therefore, we measured the zeta potential of rAAV particles at pH 7.4 and estimated their net charge from the measured electrophoretic mobility and diffusion coefficient. In addition, the outer SASA of the rAAV capsid was calculated by categorizing surface residues into hydrophobic or hydrophilic components. Figure 2 summarizes the zeta potential, net charge, and outer SASA of hydrophobic and hydrophilic amino acid residues for each rAAV serotype. All rAAV serotypes were negatively charged at pH 7.4, which is consistent with previous reports showing that rAAV particles typically exhibit negative zeta potentials near neutral pH.^39,40^ Among the tested serotypes, rAAV2 exhibited the lowest zeta potential.

**Figure 2:**
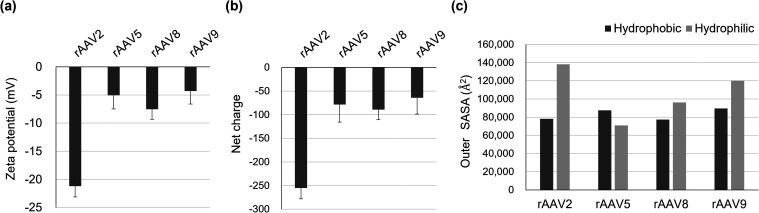
(a) Zeta potential, (b) net charge, and (c) outer SASA of hydrophobic and hydrophilic amino acid residues for rAAV2, rAAV5, rAAV8, and rAAV9. The zeta potential of rAAVs was measured in 10 mM sodium phosphate buffer (pH 7.4) containing 50 mM NaCl and 0.001% P188. Zeta potential and net charge are presented as mean ± standard deviation (*n* = 3). Replicate results are provided in Tables S1 and S2. Outer SASA values were calculated using the following PDB entries: AAV2 (PDB code: 1lp3), AAV5 (PDB code: 7kp3), AAV8 (PDB code: 2qa0), and AAV9 (PDB code: 3ux1). Surface representation of rAAV capsids showing hydrophobic and hydrophilic residues is provided in Figure S1.

The outer SASA values of hydrophobic and hydrophilic amino acid residues of rAAVs are considered to influence rAAV interactions with solid surfaces. The outer SASA of hydrophobic amino acid residues was relatively consistent among the four serotypes. In the case of rAAV5, the SASA of hydrophobic residues was higher than that of hydrophilic residues. These results reflect the low sequence homology between amino acid sequence of rAAV5 and those other serotypes.

### Contributions of Electrostatic and Hydrophobic Interactions to rAAV Adsorption

To investigate the contributions of surface properties to the nonspecific adsorption of rAAV particles, the adsorption ratios of rAAVs onto various solid surfaces were evaluated in a solvent consisting of 10 mM sodium phosphate buffer (pH 7.4) and 200 mM NaCl (Figure 3). The adsorption ratios of all rAAV serotypes on the surface of Coating Neg/PP, which was hydrophilic and negatively charged, was lower than that on the surface of untreated PP, which was hydrophobic and negatively charged. Considering that both the surfaces of untreated PP and Coating Neg/PP and rAAVs were all negatively charged, these findings suggested that hydrophobic interactions significantly contributed to the nonspecific adsorption of rAAV particles. To further examine the effect of surface charge, we compared the adsorption ratios of rAAVs on the surfaces of Coating Neg (negatively charged)/PP and Coating Pos (positively charged)/PP, both of which were hydrophilic. The adsorption ratios of rAAV2, rAAV8, and rAAV9 on Coating Pos/PP were higher than those on Coating Neg/PP, indicating enhanced adsorption to the positively charged surface via electrostatic interactions. A similar trend was observed for rAAV5, although the result was statistically insignificant.

**Figure 3:**
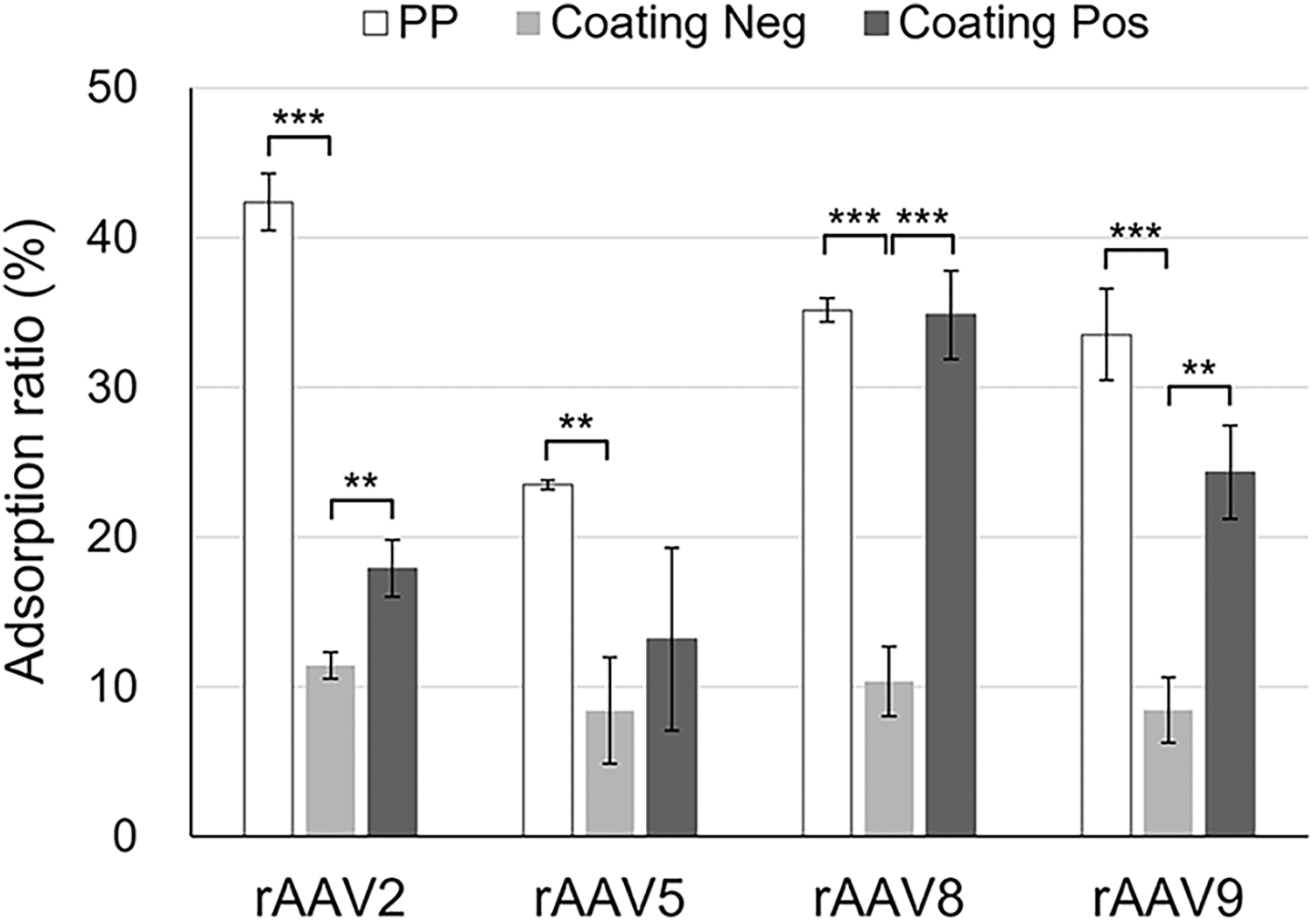
Adsorption ratios of rAAV2, rAAV5, rAAV8, and rAAV9 on the surfaces of PP, Coating Neg/PP, and Coating Pos/PP in 10 mM sodium phosphate buffer (pH 7.4) containing 200 mM NaCl. Data are presented as mean ± standard deviation (*n* = 3). Replicate results are provided in Table S3. Statistical significance was evaluated using a two-tailed unpaired Student’s *t*-test. *p < 0.05, **p < 0.01, ***p < 0.001.

To dissect the contributions of hydrophobic and electrostatic interactions to the adsorption of rAAVs, we examined the effects of ionic strength and surfactants on the adsorption ratio under three conditions: 200 mM NaCl with or without 0.001% P188 and 350 mM NaCl without the surfactant (Figure 4). In the absence of P188, adsorption ratios of rAAVs on the surfaces of Coating Neg/PP and Coating Pos/PP tended to be lower at 350 mM NaCl than at 200 mM, indicating suppression of adsorption by electrostatic shielding at the increased ionic strength. It should be noted that a similar trend was also observed on the PP surface, suggesting that electrostatic interactions also contributed to adsorption on hydrophobic surfaces. On the negatively charged surface of Coating Neg/PP, electrostatic repulsion likely occurred between the negatively charged rAAV particles and the surface, and accordingly, the adsorption ratio was expected to increase under the condition of high ionic strength because the repulsive force would be reduced. Unexpectedly, the adsorption ratios of rAAV particles decreased under the condition of high ionic strength. One possible interpretation of this result is the nonuniform distribution of surface charges on AAV capsids,^27,29,41^ which mediates adsorption via electrostatic interactions between localized positive charges on the capsid surface^42,43^ and the negatively charged solid surface. Under the condition of high ionic strength, these electrostatic interactions were weakened, leading to reduced adsorption.

**Figure 4:**
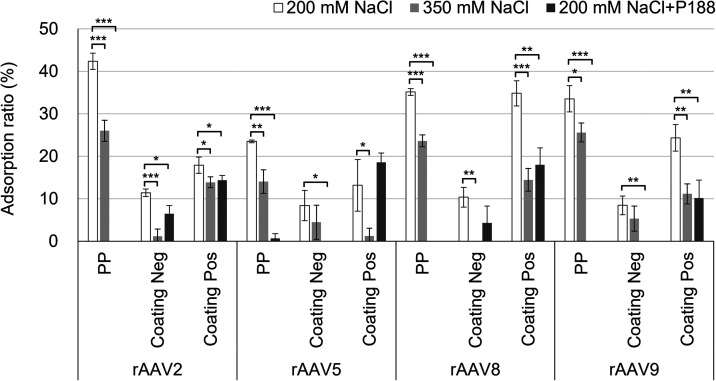
Adsorption ratios of rAAV2, rAAV5, rAAV8, and rAAV9 on the surfaces of PP, Coating Neg/PP, and Coating Pos/PP in 10 mM sodium phosphate buffer (pH 7.4) containing 200 mM NaCl, 350 mM NaCl, or 200 mM NaCl with 0.001% P188. Data are presented as mean ± standard deviation (*n* = 3). Replicate results are provided in Tables S4 and S5. Statistical significance was evaluated using a two-tailed unpaired Student’s *t* test. *p < 0.05, **p < 0.01, ***p < 0.001.

In the presence of 0.001% P188, the adsorption of rAAV particles of all serotypes on the hydrophobic PP surface was clearly suppressed. This is attributed to the preferential adsorption of the surfactant to the PP surface, which prevents rAAV particles from interacting with the PP surface.^44,45^ In the presence of the surfactant, the hydrophilic surfaces of Coatings Neg/PP and Pos/PP did not completely suppress rAAV adsorption. This is partly attributed to the insufficient adsorption of the surfactant to the hydrophilic surfaces, and therefore rAAV adsorption occurs via electrostatic interactions between rAAV and the hydrophilic surface. A recent study reported that the addition of P188 had a variable effect on rAAV adsorption depending on the surface material and rAAV serotype (rAAV8 vs rAAV9). While it was effective on plastic surfaces, it showed little effect on glass,^10^ indicating its limited ability to inhibit rAAV adsorption to hydrophilic surfaces.

### Construction of an Adsorption Model Using Multiple Regression Analysis

The above findings indicated that both hydrophobic and electrostatic interactions contributed to the nonspecific adsorption of rAAV particles to solid surfaces. We then performed multiple regression analysis to construct a predictive model for the nonspecific adsorption of rAAVs. This analysis used the following explanatory variables: contact angle and zeta potential of each solid surface and outer SASA of rAAV particles from adsorption tests conducted under 24 surfactant-free conditions. The contact angle and zeta potential of solid surfaces used in the regression analysis were measured under the solvent conditions of the adsorption tests, and the data set is summarized in Table S6. The absolute value of the zeta potential decreased when the solvent was changed from 1 mM KCl to sodium phosphate buffer containing 200 or 350 mM NaCl. Additionally, the absolute value of the zeta potential was lower at 350 mM NaCl than at 200 mM NaCl. These results suggested that increasing the ionic strength led to a compression of the electrical double layer on the solid surface.

First, a simple multiple regression model was constructed using the contact angle and zeta potential of the solid surfaces. However, the coefficient of determination (*R* ^2^) was 0.454, which was low (Figure S2­(a) and Table S7: Model S1). This result indicated that variables were insufficient to explain rAAV adsorption. As described earlier, rAAV adsorption onto the negatively charged surface of Coating Neg/PP decreased when the ionic strength was elevated, suggesting that interactions with localized positive charges on the rAAV capsid contributed to adsorption. Therefore, we modified the regression model by separating the zeta potential into positive and negative groups as independent variables. This approach enabled the model to reflect the experimental results, namely negatively charged rAAV particles adsorbed onto positively charged surfaces mainly via electrostatic attraction, and they adsorbed onto negatively charged surfaces through both global electrostatic repulsion and localized attraction. As a result, *R* ^2^ improved to 0.786, confirming that incorporating the sign of the zeta potential into the model was necessary for reliable prediction of rAAV adsorption (Figure S2­(b) and Table S7: Model S2).

Because SASA reflects the area of contact between the rAAV particle surface and solid surface, we further included the outer SASA of hydrophobic and hydrophilic amino acid residues as parameters representing the surface properties of rAAV particles. The SASA of hydrophobic residues had a negative coefficient and high *p*-value, suggesting limited relevance to the model. This is attributable to the small variation in hydrophobic SASA among the serotypes. In contrast, the significant contribution of hydrophilic SASA was confirmed because rAAV5, whose hydrophilic SASA was smaller than its hydrophobic SASA, tended to show lower adsorption ratios on PP and Coating Pos/PP surfaces compared with other serotypes. As a result, incorporating SASA values into the model increased *R* ^2^ to 0.850, leading to a more accurate predictive model (Figure 5, and Table 1). The final regression model is expressed as follows:

$$A=\beta_{0}+\beta_{1}\cdot \theta +\beta_{2}\cdot \zeta_{\mathrm{pos}}+\beta_{3}\cdot \zeta_{\mathrm{neg}}+\beta_{4}\cdot S_{\mathrm{hydrophobic}}+\beta_{5}\cdot S_{\mathrm{hydrophili}c}$$

where *A* is the adsorption ratio of rAAV particles, θ is the contact angle of the solid surface, ζ_pos_ and ζ_neg_ are the positive and negative components of the zeta potential, respectively, and *S*_hydrophobic_ and *S*_hydrophilic_ are the outer SASA of hydrophobic and hydrophilic residues on the rAAV capsid. The regression coefficients (β_0_ to β_5_), their *t*-values, and p-values are listed in Table 1, along with the symbols used for each explanatory variable.

**Figure 5:**
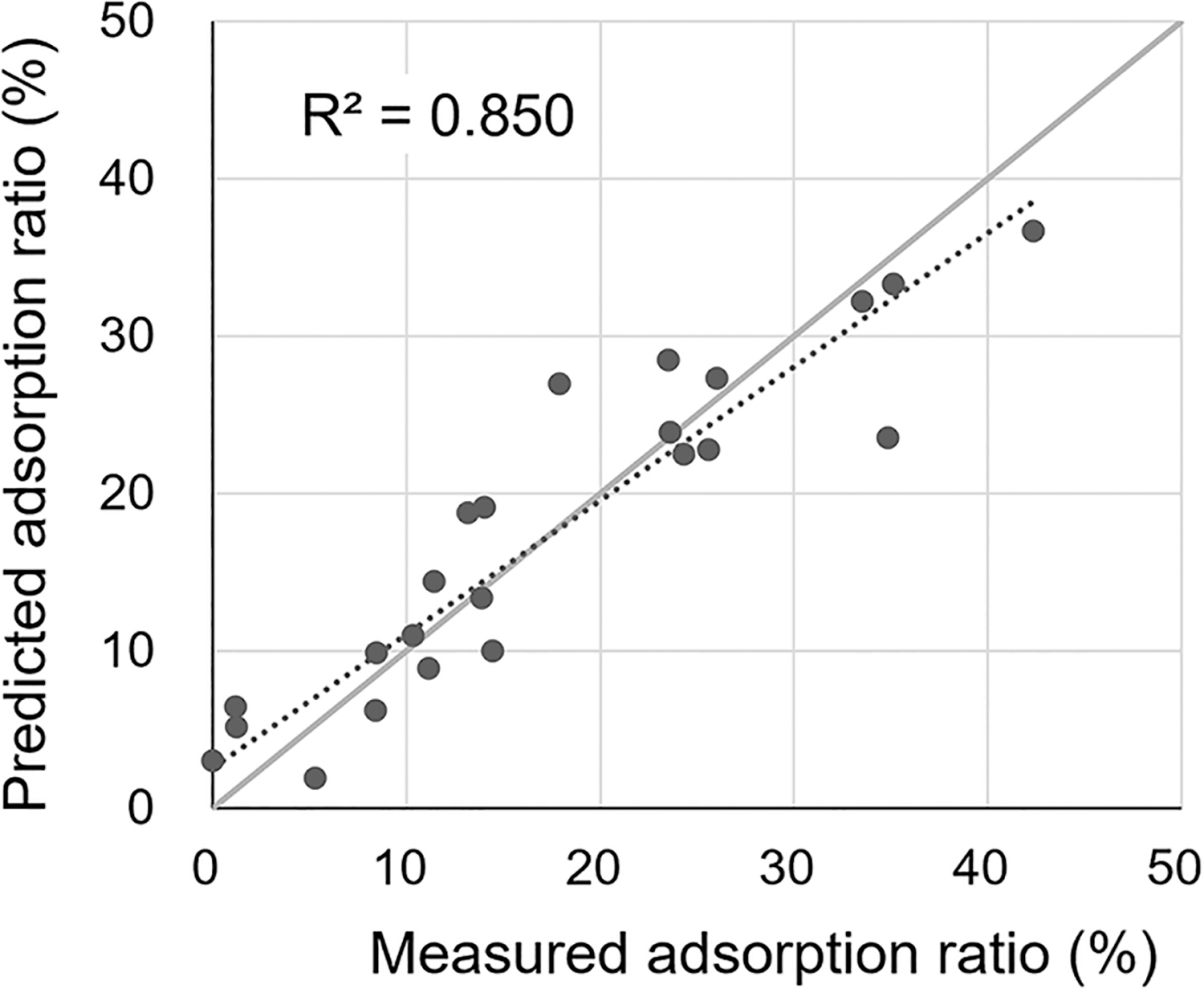
Comparison between measured and predicted adsorption ratios of rAAV particles using the constructed multiple regression model. The model includes the contact angle and absolute values of positive and negative zeta potentials of solid surfaces, as well as the outer SASA of hydrophobic and hydrophilic residues on rAAV capsids as explanatory variables. The contact angle and zeta potential of solid surfaces were measured in 10 mM sodium phosphate buffer (pH 7.4) containing 200 or 350 mM NaCl.

**Table 1:** Regression Coefficients, *t*-Values, and *p*-Values from the Constructed Multiple Regression Model

explanatory variable	symbol	coefficient	*t*-value	*p*-value
intercept		0.0041	0.022	0.983
contact angle	θ	0.0032	6.110	<0.001
zeta potential (+)	ζ_pos_	0.0247	6.496	<0.001
zeta potential (−)	ζ_neg_	–0.0042	–3.835	0.001
hydrophobic SASA	*S*_hydrophobic_	–2.587 × 10^–6^	–1.262	0.223
hydrophilic SASA	*S*_hydrophilic_	8.661 × 10^–7^	1.975	0.064

To clarify the contribution of each explanatory variable to the nonspecific adsorption of rAAVs, standardized regression coefficients of the multiple regression analysis was examined. The standardized regression coefficients were highest for the positive zeta potential (0.88), followed by the contact angle (0.72), negative zeta potential (−0.59), hydrophilic SASA (0.19), and hydrophobic SASA (−0.12) (Figure 6). As expected, electrostatic attraction between negatively charged rAAV particles and positively charged surfaces had the strongest influence on adsorption. The contribution of the contact angle further confirmed that hydrophobic interactions also played a significant role. The relatively large coefficient for the negative zeta potential supported the occurrence of rAAV adsorption to negatively charged surfaces. This suggests that adsorption cannot be fully explained by simple electrostatic attraction or repulsion. Although hydrophilic and hydrophobic SASA showed relatively small standardized coefficients, indicating their low impact on the adsorption of rAAVs to solid surfaces, they worked in opposite direction. Electrostatic interactions, which should be enhanced in hydrophilic environments, are the primarily contributor to the increased adsorption of rAAVs to surfaces, while increased hydrophobic interactions lead to decreased adsorption levels, presumably because an increase in hydrophobic SASA results in a decrease in hydrophilic SASA, which is required for electrostatic interactions and thus adsorption.

**Figure 6:**
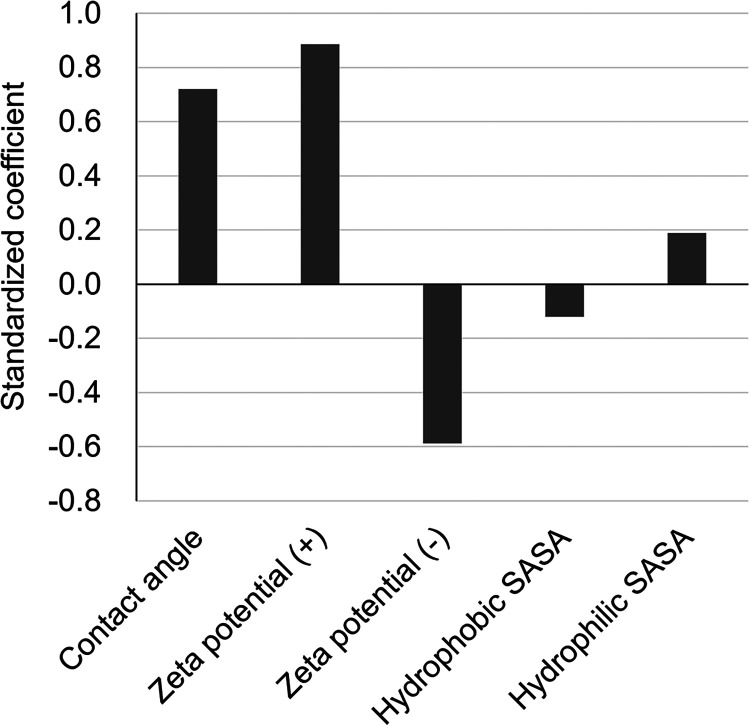
Standardized regression coefficients of explanatory variables in the constructed multiple regression model. All variables, including the adsorption ratio, were standardized using z-scores before regression analysis.

### Surface Optimization and Experimental Validation of the Predictive Adsorption Model for rAAVs

On the basis of the constructed adsorption model, we predicted that solid surfaces with a low contact angle and small absolute value of the zeta potential would effectively suppress the nonspecific adsorption of rAAV. To validate this prediction, we developed a new coating material denoted as Coating Neu. The ratio of phosphate and amine units was systematically adjusted to maintain hydrophilicity and achieve a near neutral surface charge at pH 7.4 (Figure 7(a),(b)). Adsorption tests were then conducted using PP microtubes coated with Coating Neu (Coating Neu/PP) in 10 mM sodium phosphate buffer (pH 7.4) with 200 mM NaCl. As expected, the nonspecific adsorption of all serotypes of rAAV was markedly suppressed, and the results were in good agreement with the model predictions (Figure 7(c)). Coating Neu contains phosphate and amine units, and the presence of local charge patches cannot be completely ruled out. However, due to the flexibility of the polymer chains and the potential for electrostatic interactions between phosphate and amine units in close proximity, local charge neutralization is expected. In a previous study on a similar polymer, the formation of ionic complexes between these units was reported.^46^ Moreover, the near-neutral zeta potential and suppressed rAAV adsorption observed in our experiments indicate that any charge patches that may exist do not play a dominant role in the surface interaction with rAAV.

**Figure 7:**
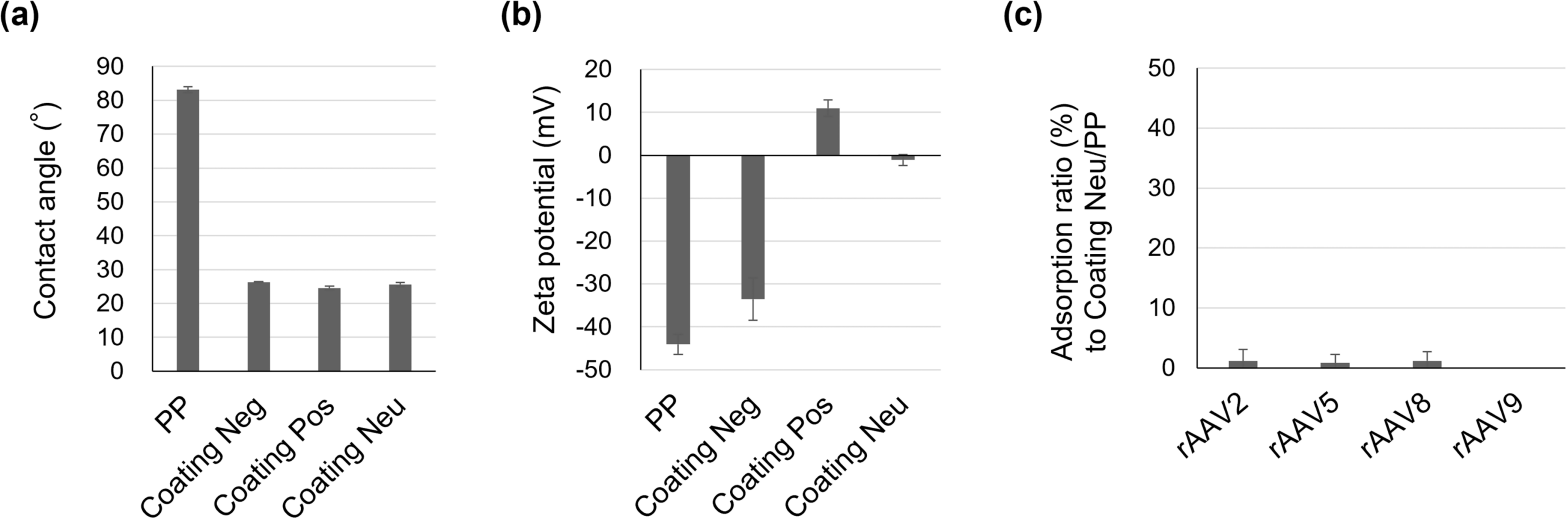
Solid surface properties and rAAV adsorption to Coating Neu/PP. (a) Water contact angle and (b) zeta potential of the surfaces of PP, Coating Neg, Coating Pos, and Coating Neu. (c) Adsorption ratios of rAAV2, rAAV5, rAAV8, and rAAV9 on Coating Neu/PP in 10 mM sodium phosphate buffer (pH 7.4) containing 200 mM NaCl. Data are presented as mean  ±  standard deviation (*n* = 3). Replicate results are provided in Table S8.

To further evaluate the versatility and practical applicability of the constructed model, additional adsorption tests were conducted under the formulation conditions of rAAV drug products (Figure 8(a)). The adsorption ratios of rAAV serotypes on Coating Neu/PP, together with untreated PP, Coating Neg/PP, and Coating Pos/PP, were evaluated. All tests were performed under surfactant-free conditions to eliminate the influence of surfactants on adsorption. The buffer compositions used for each serotype are summarized in Table 2. Adsorption ratios under these formulation conditions were predicted using the constructed model and compared with experimentally measured values. The contact angle and zeta potential of the solid surfaces measured under each formulation condition were used in the model (Table S10). As a result, the model showed high predictive accuracy, with *R* ^2^ = 0.902, which indicated that the model maintained good predictive performance even under different formulation conditions (Figure 8(b)). Importantly, Coating Neu/PP effectively suppressed the nonspecific adsorption of all serotypes. Because the pH values under all formulation conditions were similar, the surface charge of Coating Neu/PP remained near neutral, which contributed to its consistent ability to suppress nonspecific adsorption.

**Figure 8:**
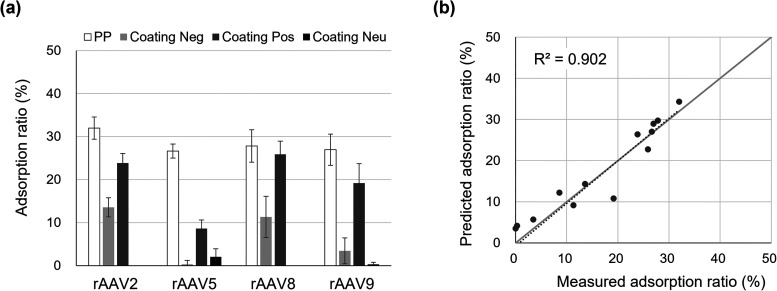
Evaluation of the predictive performance and applicability of the constructed adsorption model under formulation conditions. (a) Measured adsorption ratios of rAAV2, rAAV5, rAAV8, and rAAV9 on the surfaces of PP, Coating Neg/PP, Coating Pos/PP, and Coating Neu/PP under formulation conditions shown in Table 2. Data are presented as mean  ± standard deviation (*n* = 3). Replicate results are provided in Table S9. (b) Comparison between measured and predicted adsorption ratios calculated using the constructed regression model.

**Table 2:** Buffer Compositions Used in Adsorption Experiments Are Based on Approved Formulations of rAAV Products (rAAV2, rAAV5, and rAAV9) or a Previously Reported Formulation (rAAV8)^a^

serotype	rAAV2	rAAV5	rAAV8	rAAV9
buffer	10 mM sodium phosphate buffer	10 mM sodium phosphate buffer	20 mM Tris-HCl	20 mM Tris-HCl
pH	7.3	7.4	7.4	8.0
salt	180 mM NaCl	140 mM NaCl	100 mM NaCl	200 mM NaCl
			2 mM MgCl_2_	1 mM MgCl_2_
sugar		110 mM mannitol	2% sucrose	

a All buffers were prepared without surfactants to avoid the influence of P188 on adsorption.

These findings indicated that Coating Neu/PP, developed on the basis of the predictive adsorption model, effectively suppressed nonspecific adsorption of rAAV even under various formulation conditions, demonstrating the effectiveness of the model for surface modification leading to enhanced rAAV stability. However, if the pH of the formulation changes significantly, the zeta potential of the solid surface would also shift, which would require corresponding optimization of the surface design to reduce rAAV adsorption. This study suggests that nonspecific adsorption of rAAV is fundamentally unavoidable unless the solid surface exhibits both hydrophilicity and a near neutral surface charge.

## Conclusion

In this study, we elucidated the physicochemical factors contributing to the nonspecific adsorption of rAAV to model solid surfaces with systematically controlled hydrophilicity and surface charge. Furthermore, a predictive model for rAAV adsorption was constructed through multiple regression analysis of the adsorption of rAAV serotypes under various conditions, incorporating the physicochemical parameters of both serotypes and solid surfaces. The results revealed that both electrostatic and hydrophobic interactions played roles in nonspecific rAAV adsorption. On the basis of the constructed model, we designed a coating material (Coating Neu) with hydrophilic and neutral surface properties. Coating Neu effectively suppressed the nonspecific adsorption of various rAAV serotypes to the polymer surface, even under the formulation conditions of marketed drug products, confirming the high versatility and predictive accuracy of the model. The proposed surface modification strategy is expected to contribute to the stable manufacturing and quality control of rAAV products. Owing to its effective suppression of nonspecific rAAV adsorption, Coating Neu is a potential candidate for clinical applications, warranting further studies such as evaluation and optimization of its durability, biocompatibility, and safety.

## Supplementary Material



## Notes

Zeta potential of rAAV (Table S1); Estimated net charge of rAAV (Table S2); surface representation of rAAV capsids (Figure S1); adsorption ratios of rAAV in 200 mM NaCl buffer (Table S3); adsorption ratios of rAAV in 350 mM NaCl buffer (Table S4); adsorption ratios of rAAV in 200 mM NaCl buffer with 0.001% P188 (Table S5); data set summary for regression analysis (Table S6); comparison between measured and predicted adsorption ratios of rAAV (Figure S2); regression statistics for models S1 and S2 (Table S7); adsorption ratios of rAAV on Coating Neu/PP in 200 mM NaCl buffer (Table S8); adsorption ratios of rAAV under formulation conditions (Table S9); data set for adsorption prediction model (Table S10) (PDF)
