# Supplementary material for: Quantitative Analysis and Predictive Modeling of Nonspecific Adsorption of Recombinant Adeno-Associated Virus onto Solid Surfaces
Source: Langmuir. 2026 Feb 27;42(9):6657–66. doi: 10.1021/acs.langmuir.5c05368 (PMC12980826; doi:10.1021/acs.langmuir.5c05368)
Supplement: Supplementary file 1 [file la5c05368_si_001.pdf]

Supporting Information

Quantitative Analysis and Predictive Modeling of Nonspecific Adsorption of Recombinant Adeno-Associated Virus onto Solid Surfaces

Yuki Ueda[1,2], Risa Shibuya[2], Koichi Shibata[2], Airi Murai[2], Yasuo Tsunaka[2], Mitsuko Fukuhara[3], Susumu Uchiyama[2]*

1. Nissan Chemical Corporation, 5-1, Nihonbashi 2-Chome, Chuo-ku, Tokyo 103-6119, Japan

2. Department of Biotechnology, Graduate School of Engineering, The University of Osaka, 2-1 Yamadaoka, Suita, Osaka 565-0871, Japan

3. U-Medico Inc., 2-1 Yamadaoka, Suita, Osaka 565-0871, Japan

*Corresponding author: Susumu Uchiyama

Department of Biotechnology, Graduate School of Engineering, The University of Osaka, 2-1 Yamadaoka, Suita, Osaka 565-0871, Japan

TEL: +81-6-6879-4215, Fax: +81-6-6879-7442

Email: suchi@bio.eng.osaka-u.ac.jp

Contents

• Table S1-S10

• Figure S1 and S2

Table S1. Zeta potential of rAAV2, rAAV5, rAAV8, and rAAV9.

	rAAV2 (mV)	rAAV5 (mV)	rAAV8 (mV)	rAAV9 (mV)
n = 1	-22.3	-7.7	-7.2	-7.0
n = 2	-22.3	-4.6	-6.0	-2.7
n = 3	-19.0	-2.9	-9.5	-3.3
Mean	-21.2	-5.1	-7.5	-4.3
SD	1.9	2.4	1.8	2.3

Table S2. Net charge of rAAV2, rAAV5, rAAV8, and rAAV9.

	rAAV2	rAAV5	rAAV8	rAAV9
n = 1	-269	-119	-85	-104
n = 2	-268	-72	-70	-40
n = 3	-228	-45	-112	-49
Mean	-255	-78	-89	-64
SD	23	37	21	34

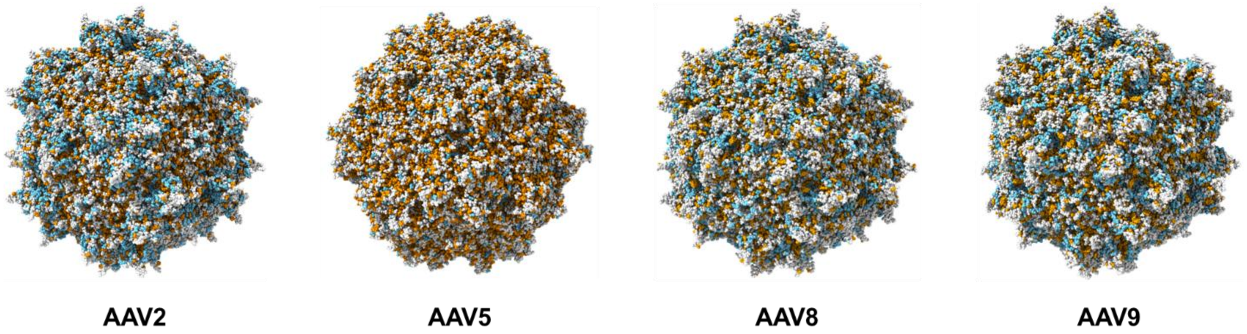

Figure S1. Surface representation of rAAV capsids showing hydrophobic residues (orange), hydrophilic residues (blue), and other residues (white). Structures were visualized using the following PDB entries: AAV2 (PDB code: 1lp3), AAV5 (PDB code: 7kp3), AAV8 (PDB code: 2qa0), and AAV9 (PDB code: 3ux1).

Table S3. Adsorption ratios of rAAV2, rAAV5, rAAV8, and rAAV9 on the surfaces of PP, Coating Neg/PP, and Coating Pos/PP in 10 mM sodium phosphate buffer (pH 7.4) containing 200 mM NaCl.

	rAAV2			rAAV5		
	PP	Coating Neg/PP	Coating Pos/PP	PP	Coating Neg/PP	Coating Pos/PP
n = 1	43%	12%	20%	24%	9%	20%
n = 2	40%	12%	16%	24%	5%	9%
n = 3	44%	10%	17%	23%	12%	10%
Mean	42%	11%	18%	23%	8%	13%
SD	2%	1%	2%	0%	4%	6%

	rAAV8			rAAV9		
	PP	Coating Neg/PP	Coating Pos/PP	PP	Coating Neg/PP	Coating Pos/PP
n = 1	36%	8%	32%	35%	9%	27%
n = 2	36%	12%	38%	30%	10%	25%
n = 3	34%	12%	34%	35%	6%	21%
Mean	35%	10%	35%	34%	8%	24%
SD	1%	2%	3%	3%	2%	3%

Table S4. Adsorption ratios of rAAV2, rAAV5, rAAV8, and rAAV9 on the surfaces of PP, Coating Neg/PP, and Coating Pos/PP in 10 mM sodium phosphate buffer (pH 7.4) containing 350 mM NaCl.

	rAAV2			rAAV5		
	PP	Coating Neg/PP	Coating Pos/PP	PP	Coating Neg/PP	Coating Pos/PP
n = 1	29%	2%	13%	17%	2%	3%
n = 2	25%	2%	14%	13%	3%	2%
n = 3	24%	0%	15%	12%	9%	0%
Mean	26%	1%	14%	14%	4%	1%
SD	2%	1%	1%	3%	4%	1%

	rAAV8			rAAV9		
	PP	Coating Neg/PP	Coating Pos/PP	PP	Coating Neg/PP	Coating Pos/PP
n = 1	22%	0%	12%	27%	9%	10%
n = 2	24%	0%	17%	26%	4%	14%
n = 3	25%	0%	14%	23%	3%	10%
Mean	24%	0%	14%	26%	5%	11%
SD	1%	0%	3%	2%	3%	2%

Table S5. Adsorption ratios of rAAV2, rAAV5, rAAV8, and rAAV9 on the surfaces of PP, Coating Neg/PP, and Coating Pos/PP in 10 mM sodium phosphate buffer (pH 7.4) containing 200 mM NaCl with 0.001% P188.

	rAAV2			rAAV5		
	PP	Coating Neg/PP	Coating Pos/PP	PP	Coating Neg/PP	Coating Pos/PP
n = 1	0%	7%	14%	2%	0%	20%
n = 2	0%	8%	16%	0%	0%	16%
n = 3	0%	4%	14%	0%	0%	20%
Mean	0%	6%	14%	1%	0%	19%
SD	0%	2%	1%	1%	0%	2%

	rAAV8			rAAV9		
	PP	Coating Neg/PP	Coating Pos/PP	PP	Coating Neg/PP	Coating Pos/PP
n = 1	0%	1%	22%	0%	0%	14%
n = 2	0%	3%	17%	0%	0%	6%
n = 3	0%	9%	15%	0%	0%	10%
Mean	0%	4%	18%	0%	0%	10%
SD	0%	4%	4%	0%	0%	4%

Table S6. Summary of the dataset used for multiple regression analysis of rAAV adsorption.

Run	Adsorption ratio	Serotype	Hydrophobic SASA (Å²)	Hydrophilic SASA (Å²)	Solid Surface	NaCl (mM)	Contact Angle (°)	Zeta Potential (mV)
1	42%	rAAV2	78,205	138,187	PP	200	83.1	-44.1
2	11%				Coating Neg/PP		26.3	-33.6
3	18%				Coating Pos/PP		24.6	+11.0
4	26%				PP	350	82.2	-22.2
5	1%				Coating Neg/PP		28.6	-12.6
6	14%				Coating Pos/PP		26.4	+5.2
7	23%	rAAV5	87,387	70,948	PP	200	83.1	-44.1
8	8%				Coating Neg/PP		26.3	-33.6
9	13%				Coating Pos/PP		24.6	+11.0
10	14%				PP	350	82.2	-22.2
11	4%				Coating Neg/PP		28.6	-12.6
12	1%				Coating Pos/PP		26.4	+5.2
13	35%	rAAV8	77,328	96,217	PP	200	83.1	-44.1
14	10%				Coating Neg/PP		26.3	-33.6
15	35%				Coating Pos/PP		24.6	+11.0
16	24%				PP	350	82.2	-22.2
17	0%				Coating Neg/PP		28.6	-12.6
18	14%				Coating Pos/PP		26.4	+5.2

19	34%	rAAV9	89,545	120,110	PP	200	83.1	-44.1
20	8%				Coating Neg/PP		26.3	-33.6
21	24%				Coating Pos/PP		24.6	+11.0
22	26%				PP	350	82.2	-22.2
23	5%				Coating Neg/PP		28.6	-12.6
24	11%				Coating Pos/PP		26.4	+5.2

Contact angle and zeta potential of solid surfaces were measured in 10 mM phosphate buffer (pH 7.4) containing 200 or 350 mM NaCl.

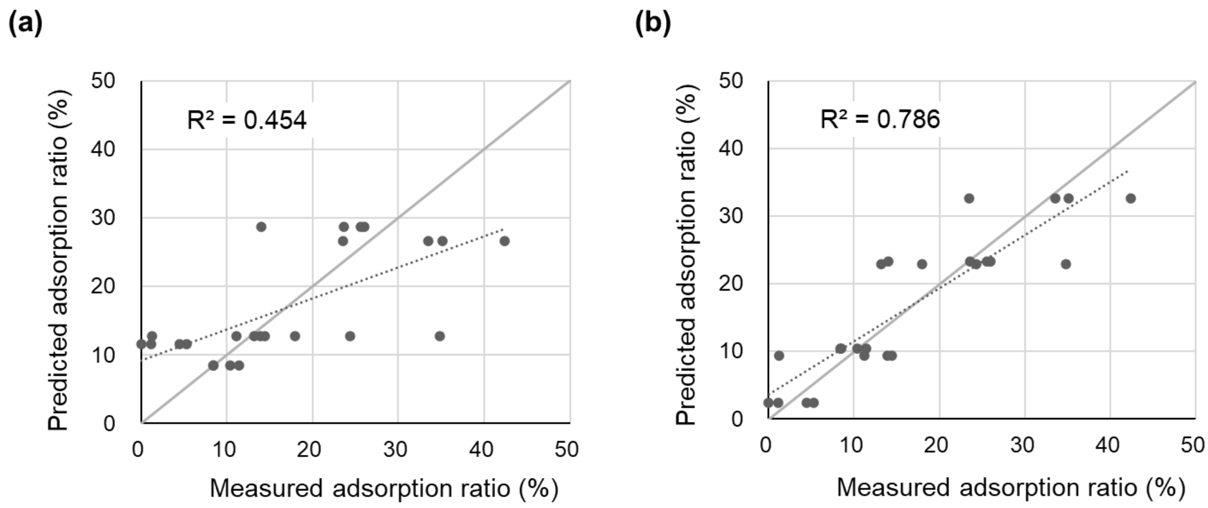

Figure S2. Comparison between measured and predicted adsorption ratios of rAAV particles using multiple regression models. (a) Model S1: multiple regression using the contact angle and zeta potential of solid surfaces. (b) Model S2: multiple regression using the contact angle and absolute values of positive and negative zeta potentials of solid surfaces as separate variables.

Table S7. Regression coefficients, *t*-values, and *p*-values from multiple regression models S1 and S2.

Explanatory Variable	Coefficient	*t*-value	*p*-value
**Model S1 (R^2 = 0.454)**			
Intercept	0.0326	0.869	0.395
Contact angle	0.0034	3.735	0.001
Zeta potential	0.0011	0.899	0.379
**Model S2 (R^2 = 0.786)**			
Intercept	-0.1188	-3.276	0.004
Contact angle	0.0032	5.399	< 0.001
Zeta potential (+)	0.0247	5.741	< 0.001
Zeta potential (-)	-0.0042	3.389	0.003

Table S8. Adsorption ratios of rAAV2, rAAV5, rAAV8, and rAAV9 on Coating Neu/PP in 10 mM sodium phosphate buffer (pH 7.4) containing 200 mM NaCl.

	rAAV2	rAAV5	rAAV8	rAAV9
n = 1	3%	0%	1%	0%
n = 2	0%	0%	0%	0%
n = 3	0%	3%	3%	0%
Mean	1%	1%	1%	0%
SD	2%	1%	2%	0%

Table S9. Adsorption ratios of rAAV2, rAAV5, rAAV8, and rAAV9 on the surfaces of PP, Coating Neg/PP, Coating Pos/PP, and Coating Neu/PP under formulation conditions shown in Table 2.

	rAAV2				rAAV5			
	PP	Coating Neg/PP	Coating Pos/PP	Coating Neu/PP	PP	Coating Neg/PP	Coating Pos/PP	Coating Neu/PP
n = 1	35%	16%	26%	0%	28%	0%	9%	2%
n = 2	30%	14%	23%	0%	28%	1%	11%	0%
n = 3	31%	11%	22%	0%	25%	0%	7%	4%
Mean	32%	14%	24%	0%	27%	0%	9%	2%
SD	3%	2%	2%	0%	2%	1%	2%	2%

	rAAV8				rAAV9			
	PP	Coating Neg/PP	Coating Pos/PP	Coating Neu/PP	PP	Coating Neg/PP	Coating Pos/PP	Coating Neu/PP
n = 1	32%	11%	29%	0%	23%	6%	19%	0%
n = 2	25%	16%	23%	0%	27%	4%	15%	0%
n = 3	26%	6%	25%	0%	30%	0%	24%	1%
Mean	28%	11%	26%	0%	27%	3%	19%	0%
SD	4%	5%	3%	0%	4%	3%	5%	0%

Table S10. Dataset used in the constructed regression model to predict adsorption ratios.

Run	Adsorption ratio	Serotype	Hydrophobic SASA (Å^2)	Hydrophilic SASA (Å^2)	Solid surface	Contact Angle (°)	Zeta Potential (mV)
1	32%	rAAV2	78,205	138,187	PP	82.4	-38.8
2	14%				Coating Neg/PP	27.7	-32.3
3	24%				Coating Pos/PP	27.1	+10.4
4	0%				Coating Neu/PP	25.6	-7.9
5	27%	rAAV5	87,387	70,948	PP	86.6	-38.0
6	0%				Coating Neg/PP	25.0	-29.6
7	9%				Coating Pos/PP	25.7	+8.2
8	2%				Coating Neu/PP	24.4	-1.9
9	28%	rAAV8	77,328	96,217	PP	83.3	-35.3
10	11%				Coating Neg/PP	27.5	-28.2
11	26%				Coating Pos/PP	26.2	+10.4
12	0%				Coating Neu/PP	26.2	-6.1
13	27%	rAAV9	89,545	120,110	PP	84.4	-35.3
14	3%				Coating Neg/PP	26.4	-23.3
15	19%				Coating Pos/PP	26.0	+6.1
16	0%				Coating Neu/PP	23.8	-3.8

Contact angle and zeta potential of solid surfaces were measured under buffer conditions shown in Table 2.
